# The Impact of Ethical Leadership, Commitment and Healthy/Safe Workplace Practices toward Employee Attitude to COVID-19 Vaccination/Implantation in the Banking Sector in Lebanon

**DOI:** 10.3390/vaccines10030416

**Published:** 2022-03-10

**Authors:** Samira Kabbani, Silva Karkoulian, Puzant Balozian, Sandra Rizk

**Affiliations:** 1 Adnan Kassar School of Business, Lebanese American University, Beirut 1102-2801, Lebanon; samira.kabbani@lau.edu (S.K.); skarkoul@lau.edu.lb (S.K.); puzant.balozian@lau.edu.lb (P.B.); 2 Department of Natural Sciences, Lebanese American University, Beirut 1102-2801, Lebanon

**Keywords:** ethical leadership, commitment, healthy/safe workplace, COVID-19 vaccination, quantum dot technology, on-patient vaccine records

## Abstract

This study investigates the effect of ethical leadership, commitment and healthy/safe workplace practices toward employee COVID-19 vaccination. In addition, this study examines the perception of employees from technological intrusive vaccination of chips or quantum dot. In our research, we adopted the social exchange theory as its theoretical framework. Moreover, an online questionnaire was distributed to employees working in the banking sector in Lebanon during the COVID-19 pandemic. In total, 244 bankers completed the survey. Data was analyzed by SPSS statistical software version 26 and SmartPLS to test the relationship between the variables. The results generated showed a positive relationship between ethical leadership, commitment, and safety influencing employees to accept vaccination but not necessarily technological intrusive vaccination (chip or quantum dot). We suggest that organizations should influence leaders to enhance proper behaviors and attitudes to create a healthy, safe, and ethical culture that consequently increases employees’ commitment. Finally, this study recommends future researchers to investigate the topic of COVID-19 vaccination and test other employees’ perception from different industries and countries.

## 1. Introduction

The viral infection “COVID-19” was first detected at Wuhan City, China on 31 December 2019 [1]. The reason behind this disease was novel coronavirus (COVID-19), that was declared as a pandemic by the World Health Organization (WHO) on 11 March 2020 [2]. Despite the lockdowns and precautionary measures, the virus spread rapidly in China and other countries around the world causing numerous infections and deaths. COVID-19 spread fast around the world compared to H1N1 and SARS diseases that were effectively controlled much faster [3]. As of 26 January 2022, the statistics revealed 360,161,697 infected cases, more than 5,636,851 deaths, and 285,046,642 recovered patients from all countries [4].

To prevent COVID-19 spread, almost all countries implemented proposed recommendations by the World Health Organization to control this crisis [3]. Some countries adhered to certain measures, such as closing borders, suspending organizations’ work, cancelling gatherings, wearing masks, social distancing, and movement restrictions. Consequently, such measures caused a detrimental impact on the world economy.

On 15 March 2020 the increase of infected citizens with COVID-19, obliged the government to implement a lockdown to decrease the spread of the pandemic in Lebanon [5]. These decisions varied between lockdown curfew, odd and even plate numbers and other restrictions to limit the impact of this virus [5]. Although these decisions yielded fruitful outcomes in diminishing the overall number of infections, this situation changed a few months later where the number of COVID-19 cases started to rise significantly in 2020 [5]. Accordingly, the government was forced to implement a second lockdown to contain the health situation in Lebanon [5]. Hence, on 15 March 2020 the total number of cases exceeded 418,000 and the total deaths were counted to 5380 [5].

During the initial stages of the pandemic, conspiracies began to circulate around the nature of this virus. Most of the skepticism was around the contents of the vaccine. A plethora of people were under the impression that upon receiving the vaccine, they would find themselves implanted with a microchip that tracks their every move [6]. It was said that these microchips will be secretly implanted in our bodies making “people’s arms magnetic” and letting some governments track those who are vaccinated [7]. Some people began claiming that the coronavirus pandemic was only a plan to “implant trackable microchips” for people [8]. This indicated that before its release, the vaccine had already instigated a negative manifestation in peoples’ thought processes.

In order to better serve our society and to save lives by persuading as many vulnerable persons as possible that the benefits of the vaccine outweigh its costs, we wanted to (1) find some work-related antecedents of vaccine acceptance, to (2) shed light on the reasons behind the harmful vaccine hesitancy conspiracy theories and to (3) give recommendations to the scientific community related to vaccine-linked technology advancements. Since the prominent MIT Technology Review magazine suggests that behind every conspiracy theory there might be a small kernel of truth [9], we investigated the source idea of the “tracking aspect” of the vaccine in order to bring clarification to a confused society. Our research led us to a scientific publication about vaccine enabled quantum dots technology [10].

Quantum Dots (QD) are biodegradable colors that are surmounted to a vaccine enabled needle. This advancement in technology can be summarized as a “specialized invisible dye, delivered along with a vaccine, could enable “on-patient” storage of vaccination history to save lives in regions where paper or digital records aren’t available” [11]. Since the QD technology is new, we strived to use a proxy variable established in the medical scientific literature in order to (1) use a language understood by our sample respondents (very few people know about QD, which might have skewed our research), and to (2) measure perceptions of people toward having vaccination records/history “on-patient”. That proxy variable was the perceptions toward the “microchip”, adapted from instrument items found in a high-quality journal [12]. Although the size of a rice microchip radio frequency (RFID) technology and its deployment for animal and human use are not new, we emphasize that the use of that variable in our study was not due to a distorted idea about the existence of microchip-surmounted COVID vaccines, but was employed only on the basis of (a) the closest proxy we could find related to quantum dot technology without confusing the respondents with technological jargon they could not understand, and on the basis that, (b) to the best of our knowledge, it is the foremost variable that relates to storing vaccine information below the skin’s surface and that is concurrently validated in prominent medical journals.

Thus, we were further interested to assess how negative perceptions of the vaccine would stunt its acceptance in an educated portion of society. In addition, we wanted to test if leaders can influence their employees to receive (a) vaccination and (b) on-patient vaccination record. If these leaders can drive people to change their minds as to whether or not they will take the vaccine, that would speak volumes on the structure of leadership, as well as its ethics and would further the cause of increasing vaccination records in the society.

After one year from the spread of COVID-19, the World Health Organization announced in early December 2020 the introduction of vaccination programs against the pandemic including 13 different types of vaccination such as Pfizer and AstraZeneca, among others (2021). A survey was conducted in Lebanon to test people’s perception of COVID-19 vaccination revealed that 40% of participants strongly disagreed with receiving the vaccination [5]. In this sense, this research focuses on testing the impact of ethical leadership, commitment, healthy/safe workplace practices toward employee acceptance of COVID-19 vaccination in the Lebanese banking sector. In addition, the employee perception toward “on-patient” vaccine record was also evaluated.

## 2. Materials and Methods

The survey was designed for voluntary participants, in the English language, to take about 10 min for completion, and all the questions were mandatory. For easier data collection after COVID-19 inflation, a softcopy format for the survey was conducted through “Google Form” (https://www.google.com/forms/about/, accessed on 21 January 2022) for data gathering. The survey included six sections consisting of five scales: Consent Form, Demographic Data, and several sections that included 50 questions related to the following scales: Ethical Leadership, Commitment, Healthy/Safe Workplace, COVID-19, and Microchips. These scales were tested for their validity and reliability in previous studies.

Based on previous literature reviews, the survey was conducted based on multiple studies that includes several scales: Ethical Leadership [13,14], Commitment [15,16], Workplace Safety [17,18], and employees’ attitudes toward COVID-19 Vaccination [19,20], and Microchips [12]. The respondents provided their feedback to the questions through the Likert Type format from 1 to 5 in which “1” means strongly disagree and “5” means strongly agree.

After receiving the Institutional Review Board (IRB)’s approval for the survey on 24 November 2020, data collection from participants was initiated. Employees in the Lebanese banking sector received an Email stating the purpose of the study with a link to participate in the survey. Interested candidates accessed the link that guides them to a Google Form Page that includes the survey content: consent from, demographics, and then 50 questions related to “Ethical Leadership, Commitment, Healthy/Safety Workplace, and COVID-19 Vaccination”.

The total number of participants collected was 246. However, 2 responses from the total collected were excluded due to incomplete answers by participants. Therefore, the data obtained from 244 respondents were used for the analysis through SPSS software (https://www.ibm.com/analytics/spss-statistics-software/, accessed on 21 January 2022) and SMART https://www.kvk.nl/advies-en-informatie/innovatie/smart-doelen-stel-je-zo/, accessed on 21 January 2022).

## 3. Results

The data reporting the respondents’ demographics is presented in Table 1. The results show that 129 of our respondents are females (52.9%) and 115 are males (47.1%). This indicates gender equality since percentages are almost equated. Concerning age, most of the respondents belonged to age bracket between 26 and 44 years with 63.5%, followed by participants from the age group between 45 and 56 years with 19.7%, then above 57 years with 8.6%, and finally below 25 years with 8.2%. Therefore, we can conclude that the majority of our respondents are young and middle aged. Moreover, the majority of respondents (51.6%) were in the marital status of married with children. Single participants represented 34.4%, while married with no children 10.2%, divorced respondents represented 3.3%, and widowed were the fewest (0.4%). With respect to the educational level, 60.2% of respondents were master’s degree holders, 30.7% had undergraduate degrees, 6.6% had other professional qualifications, and finally 2.5% had doctorate degrees. From these results, we can conclude that the majority of respondents are highly educated. As for years of work experience, 103 respondents had more than 15 years of experience (42.2%), 51 respondents with 11 to 15 years of experience (20.9%), 44 respondents with 6 to 10 years of experience (18%), followed by 40 respondents with 1 to 5 years of experience (16.4%), and finally 6 respondents with less than one year of experience (2.5%). This indicates that we had both experienced and middle-experienced respondents who participated in this questionnaire, noting that we had more skewness toward high years of experience. Concerning organizational level, most respondents belong to operational management with 34.8% of the total, then non-management with 31.1%, followed by middle management with 21.3%, and finally senior management with 12.7%. Thus, we can conclude that the responses are reliable because there are 29 of them collected from all levels of the organization (Entry, Middle, and Senior). In regards to participants’ health status with respect to COVID-19, 80.7% of the respondents did not get infected with COVID-19, 16% were infected, and 3.3% were currently infected while completing the questionnaire.

The measurement model examination includes internal reliability, convergent and discriminant validity of the measurement items. Table 2 illustrates the internal consistency reliabilities. All constructs but one scored above 0.7, which is the recommended threshold of Cronbach’s alpha [21]. However, the composite reliability exceeds the required threshold of 0.7 [22].

The items displayed acceptable measures for convergent and discriminant validity. Convergent validity is achieved when the average variance extracted (AVE) of each construct is above 0.5 [22]. All the constructs in our model demonstrated an AVE record above 0.5. In addition, the discriminant validity is satisfied if the square root of the AVE of each construct in the diagonal is higher than the variance of all the other constructs [23]. Table 3 illustrates the discriminant validity measures. Finally, discriminant and convergent validity was further tested in the loadings and cross loading.

In general, item loadings higher than 0.6 on their related factors are considered acceptable [24]. Table 4 displays the outer weights of our model. As shown below, the loadings in the constructs met the required threshold as anticipated, and they were higher than the loadings across constructs. Therefore, the psychometric properties and the scales show acceptable convergent and discriminant validity.

Each item has an outer loading that influence the overall model. If the weights of the outer loading are significant then they should be kept in the study, however if they were not significant then they should be consulted. All overall scores should be above 0.5, if the loading is higher than 0.5 then it must be kept. However, if the loading is less than 0.5 there are two possible solutions. First, if the loading is not significant then we remove this item from research, or if the item is significant then we have the option to remove it or keep it in the research. In our data analysis, some items had an insignificant outer loading, therefore we removed them from the research.

**Figure 1:**
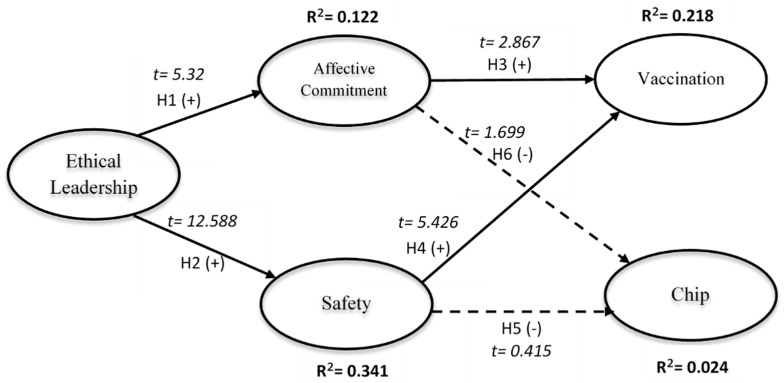
Structural Model that illustrates variable relationships in this study.

The structural model predicts the path coefficients, and the coefficient determination (R²). The path coefficient measures the strength of the relationship between dependent and independent variables, while the coefficient of determination (R²) is the amount of variance encountered by independent variables. Figure 1 illustrates the paths and the prediction level of the model. Both ethical leadership and commitment predict around 12.2%, ethical leadership and safety predicts 34.1%, chips measured 2.4% and vaccination recorded 21.8%.

The structural model supported four of the six hypothesized relationships. As shown in Figure 1, ethical leadership has a significant positive influence on affective commitment (H1: ß = 0.349, *p* < 0.001). The path of ethical leadership to safety is also supported (H2: ß = 0.584, *p* < 0.001). The path of affective commitment to vaccination was significant (H3: ß = 0.203, *p* < 0.004), while affective commitment to microchip is not significant (H6: ß = 0.14, *p* < 0.09). Concerning path of safety to vaccination the result was significant (H4: ß = 0.354, *p* < 0.001), while to chip it was not significant (H5: ß = 0.03, *p* < 0.678).

## 4. Discussion

This study utilizes social exchange theory to explore the impact of ethical leadership, commitment, healthy/safe workplace practices toward employees’ attitudes to COVID-19 vaccination and microchip implantation. 

Our first hypothesis states that ethical leadership is positively related to commitment based on the findings of previous literature reviews. The results obtained support a relationship between the variables; this indicates that ethical leadership positively impacts employee commitment. The study’s second hypothesis, stating that ethical leadership is positively related to an employee’s likelihood to enjoy a healthy and safe workplace, was also supported. The third hypothesis that commitment is significantly related to COVID-19 vaccination revealed a positive relationship. The fourth hypothesis that employees’ need for a healthy and safe workplace is positively related to COVID-19 vaccination was also supported. However, the fifth hypothesis stating that employees’ need for a healthy and safe workplace is significantly related to microchip implantation showed that there is no relation between the variables. Finally, the last hypothesis stating that commitment is related to microchip implantation also showed that there is no association between the variables.

Based on these outcomes, we have a basis to claim that ethical leadership, commitment, and healthy/safe workplace practices are essential in employee acceptance of COVID-19 vaccination in the banking sector in Lebanon. The present research clearly indicates that if ethical behaviors and a safe climate are created in an organizational culture, this would create an attachment and feeling of loyalty to the organization. These working conditions significantly influenced employees to accept the COVID-19 vaccination compared to several literature reviews that argued that more than 50% of the population in the United States and United Kingdom are against vaccination [25,26,27].

Ethical leaders are essential to influence, direct, motivate and encourage ethical behaviors as well as attitudes in organizations. Consequently, top management should ensure the employment of ethical leaders to generate an ethical culture for employees. In addition, organizations should implement proper policies and awareness training related to safety measures to keep employees safe in the workplace. Additionally, employee commitment to the workplace increases employee productivity and loyalty in addition to decreasing turnover and absenteeism. In summary, all these factors contribute to employees’ positive perception of the COVID-19 vaccine and hence their willingness to receive COVID-19 vaccination.

It is worth noting that, although the banking sector in Lebanon is facing a critical condition, and banks are terminating many employees, employees were still willing to get the COVID-19 vaccination. The contrary would be expected with all the working circumstances employees are facing in the banking sector in Lebanon. Employees are not expected to be committed to their employers and reject to receive COVID-19 vaccination. However, the results revealed that, with the contribution of ethical leadership behaviors, employee commitment, and by maintaining healthy and safe workplace practices, organizations can positively enhance the willingness of employees to receive vaccination.

Regarding microchip implantation, the results revealed that there is no relationship between the variables ethical leadership, commitment, and healthy/safe practices with the acceptance of the employees to implant microchips under their skin’s surface. This indicates that people still mistrust intrusive medical record-keeping technologies.

This study is a pioneer in literature because it tackles all variables together with COVID-19 vaccination and microchip implantation. Some previous articles tackle the issue of vaccination and microchip implantation, but they are considered minimal. Therefore, the combination of the variables is novel and differs from previous literature. In previous articles, authors concluded that employees in the medical services refused to get vaccination due to religious or medical concerns. Sometimes employees in the medical field were forced to receive the vaccination otherwise they would be terminated. However, in our study we were targeting employees from the banking sector and how variables such as ethical leadership, commitment, health and safe workplace practice might affect their perception of receiving COVID-19 vaccination and under the skin vaccine record. We were able to demonstrate that the tested variables influence employees to accept the former, while not accepting the latter.

One other contribution of this study is a finding that is relevant to medical, scientific and technology research communities; people might not easily accept intrusive technologies like storing medical/vaccine information under the skin via quantum dots. This could greatly impact future medical researchers especially if another virus or pandemic would arise that might lead scientists to improve technologies related to registering vaccine records on patients. If this type of research, its purpose and scope are not clearly communicated to the populace, it might lead to unnecessary vaccine hesitation among them and might give rise to needless and harmful conspiracy theories. Thus, we suggest better communication, and increased awareness about the reasons and the importance (if any) of such intrusive technologies in order not to intervene with the vaccination efforts. Better yet, detaching such on-patient record keeping technologies from vaccine efforts might yield better vaccination results.

## 5. Limitations and Future Research

This research study faced three limitations. First, the number of participants in the survey was expected to be higher. Even though we distributed the survey’s link to a large number of employees in the banking sector in Lebanon only 244 employees filled the survey. In this sense, we can conclude that banking sector employees are not motivated or committed to participate in surveys especially ones where their employment status is at stake. Second, the data collected were homogenous because the majority of banking sector employees in Lebanon are relatively educated, young, and married. According to the Association of Banks in Lebanon (Annual Report 2019), the banking sector in Lebanon includes a high level of educated, young, and married employees with more than 81% of employees holding a university degree, 58.4% of employees are below 40 years old, and married employees are 65.6% of the population. [28]. Third, very few literature reviews were found studying the topic of COVID-19 and COVID-19 vaccination with our tested variables. COVID-19 emerged in late 2019, therefore it is still considered a fresh topic with very few researchers who have examined this issue.

Therefore, future research should focus on investigating the topic of COVID-19 vaccination more and test other employees’ perceptions from different industries and countries. In addition, future researchers could also examine other variables such as job satisfaction, engagement with COVID-19 vaccination, since other variables might also affect employees to accept or reject COVID-19 vaccination, quantum dot (QD) technologies, and vaccine enabled QDs. In this sense, it is worthy to investigate the effect of other variables on our dependent variables.

## 6. Conclusions

To conclude, this paper studied the impact of ethical leadership, commitment, and healthy/safe workplace practices toward employees’ attitudes to COVID-19 vaccination and on-patient vaccine record keeping. We found that ethical leadership, commitment, and healthy/safe workplace practices impact employee behavior and decision to accept COVID-19 vaccination. However, concerning intrusive vaccine record-keeping technologies, the results revealed that there is no influence from the variables to affect employee attitude to keep such records in their bodies. Thus, it is recommended that organizations encourage leaders to behave ethically, promote a healthy and safe environment so employees would become more ethical, loyal and committed to their work. In this sense, employees will be more motivated to accept the vaccination and that will positively impact their well-being in turn. However, even ethical leadership and pleasant work environment are not powerful enough antecedents to accept on-patient intrusive vaccine record-keeping technology, probably because employees still find it unsecure and unsafe with respect to their beliefs and perceptions.

## Figures and Tables

**Table 1:** Summary of Respondents Demographics, *n* = 244.

Demographic Variable	Respondents	
	**Number**	**Percentage**
**Gender**		
*Male*	115	47.1%
*Female*	129	52.9%
**Age**		
*57–75 Years*	21	8.6%
*45–56Years*	48	19.7%
*26–44 Years*	155	63.5%
*25 Years & Less*	20	8.2%
**Marital Status**		
*Single*	84	34.4%
*Married, No Children*	25	10.2%
*Married, With Children*	126	51.6%
*Divorced*	8	3.3%
*Widow*	1	0.4%
**Educational Level**		
*Undergraduate Degree*	75	30.7%
*Master’s Degree*	147	60.2%
*Doctorate*	6	2.5%
*Other Professional Qualifications*	16	6.6%
**Years of Work Experience**		
*Less than a year*	6	2.5%
*1–5*	40	16.4%
*6–10*	44	18%
*11–15*	51	20.9%

**Table 2:** Reliability measures.

Construct	AVE	Composite Reliability	Cronbach’s Alpha
**Ethical Leadership**	0.778	0.875	0.714
**Affective Commitment**	0.786	0.88	0.735
**Health & Safety**	0.649	0.902	0.865
**Vaccination**	0.616	0.828	0.69
**Microchip**	0.875	0.933	0.86

**Table 3:** Discriminant Validity Measures.

Discriminant Validity	Microchip	Affective Commitment	Safety	Ethical Leadership	Vaccination
**Microchip**	0.935				
**Affective Commitment**	0.151	0.887			
**Safety**	0.081	0.359	0.805		
**Ethical Leadership**	0.1	0.349	0.584	0.882	
**Vaccination**	−0.014	0.331	0.427	0.375	0.785

**Table 4:** Outer Loading Testing Results.

Outer Loadings	Microchip	Affective Commitment	Safety	Ethical Leadership	Vaccination
Microchip 1	0.955				
Microchip 2	0.915				
Affective Commitment 1		0.848			
Affective Commitment 2		0.924			
Safety 1			0.776		
Safety 2			0.834		
Safety 3			0.82		
Safety 4			0.869		
Safety 5			0.72		
Ethical Leadership 1				0.873	
Ethical Leadership 2				0.891	
Vaccination 1					0.843
Vaccination 2					0.783
Vaccination 3					0.725

## Data Availability

Not applicable.
